# Advancing a standardised approach to onchocerciasis elimination mapping

**Published:** 2019-05-13

**Authors:** Louise Hamill, Becks Hill, Alex Pavluck, Philip Downs

**Affiliations:** 1 Global Technical Lead, Onchocerciasis and lymphatic filariasis; 2 Project Director; 3 Senior Technical Health Systems Director; 4 NTD Technical Director NTD Directorate, Sightsavers, 35 Perrymount Road, Haywards Heath, UK.

**The focus in onchocerciasis is shifting from the control of disease to the elimination of transmission. Completing onchocerciasis elimination mapping is key.**

Onchocerciasis, also known as river blindness, is classified as a neglected tropical disease. It causes visual impairment and blindness, stigmatising skin disease and severely debilitating itching. It was estimated in 2017 that there were 20.9 million people infected with onchocerciasis, of whom 14.6 million were suffering from skin disease and 1.15 million were visually impaired or blind.^1^

The donation of the medicine ivermectin (Mectizan^®^) by Merck & Co., Inc. (which operates as MSD outside of the United States and Canada), and the subsequent reductions in disease prevalence, has seen a shift in focus from control of clinical disease to elimination of disease transmission. There are still 205 million people living in known endemic areas, although the risk of infection to most people is low, as long as Mectizan distribution continues.^2^ Elimination of onchocerciasis transmission will ensure that this risk is removed for good, permanently protecting the health and eyesight of millions of people.

Previous mapping efforts focused on identifying the moderate to high endemic areas for priority treatment. In the context of elimination, however, low endemic areas (classified as hypo-endemic areas during previous surveys) must also be identified. Treatment must be instigated in any such area where there is ongoing transmission.

Elimination of onchocerciasis can be achieved if it is possible to increase the rate at which onchocerciasis elimination mapping is completed. In collaboration with ministries of health, WHO/ESPEN (the expanded special programme to eliminate NTDs in Africa and in Yemen), the Bill and Melinda Gates Foundation, Sightsavers and other partners have begun a series of pilot studies to test and refine the tools and strategies that will be needed for OEM. The overall aim is to operationalise and scale-up the draft OEM protocols outlined by the WHO Onchocerciasis Technical Advisory Subgroup (OTS), and to provide data and learning to support their refinement and wider use.

The first phase of OEM pilot studies were successfully completed in 2018 in Ghana and Nigeria. The studies verified onchocerciasis endemicity status in several important implementation units (the administrative level at which each country decides mapping should be conducted) and provided opportunities to test and validate the proposed protocol and data flow system. Best practices were developed and tested in relation to the operational roll-out of OEM, including field-level data collection, technical and epidemiological support, and quality control (embedded in every process). A comprehensive training package for mapping teams, and a framework for preparatory desk-based data reviews, were also developed and tested. Ministries of health will have access to real-time information on OEM as it is conducted. They will receive standardised outputs from the electronic data capture platform, which will allow them to integrate validated data into national databases and make it easier to report to WHO/ESPEN. Detailed cost analysis is underway to develop accurate cost estimations at the implementation unit level.

**Figure:**
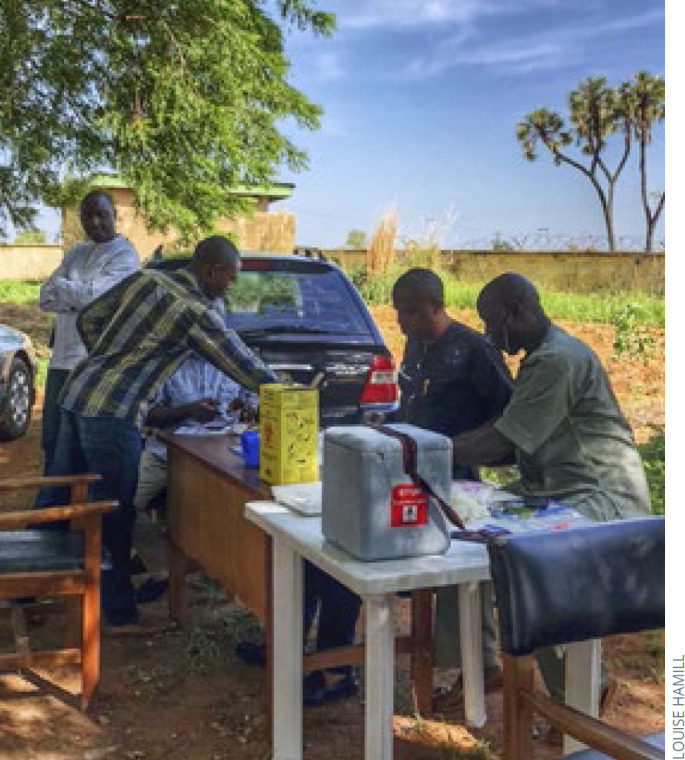
The OEM survey team get ready to screen community members in Sokoto. NIGERIA

The necessary preparatory work is underway for the final part of the OEM pilot; to conduct mapping in Mozambique in mid-2019 in several implementation units. Findings from the latest WHO OTS meeting in February 2019 are currently being included in planned activities in Mozambique. Due to the devastating impact of cyclone Idai, we are closely monitoring the situation and will work with our Mozambique partners to assess the most appropriate time for completion of this work.

On completion, the consortium will be able to offer national programmes and partner organisations in the remaining countries in need of OEM a fully piloted, costed approach to operationalising the OEM protocols outlined by the OTS on a quality-assured online platform. We anticipate that these countries will require close technical guidance from WHO/ESPEN to assist with the prioritising of areas to be mapped.

Integral to the successful roll-out of OEM pilots to date has been ensuring close ties, collaboration and coordination with partners, key stakeholders and experts.

These pilot studies are an essential step in facilitating the rapid expansion and acceleration of OEM while ensuring rigorous data quality standards are maintained.
